# Electrospun Polylactide/Natural Rubber Fibers: Effect Natural Rubber Content on Fiber Morphology and Properties

**DOI:** 10.3390/polym13142232

**Published:** 2021-07-07

**Authors:** Yulia Tertyshnaya, Svetlana Karpova, Maksim Moskovskiy, Aleksey Dorokhov

**Affiliations:** 1 Department of Biological and Chemical Physics of Polymers, Emanuel Institute of Biochemical Physics, Russian Academy of Sciences, 4 Kosygina Str., 119334 Moscow, Russia; karpova@sky.chph.ras.ru; 2 Federal Scientific Agroengineering Center VIM, 1st Institutskiy Proezd, 5, 109428 Moscow, Russia; maxmoskovsky74@yandex.ru (M.M.); vim@vim.ru (A.D.)

**Keywords:** polymer fibers, polylactide, natural rubber, non-woven fiber, electrospinning, interfacial interactions, crystalline phase, physical properties

## Abstract

Non-woven polylactide-natural rubber fiber materials with a rubber content of 5, 10 and 15 wt.% were obtained by electrospinning. The thermal, dynamic, and mechanical properties of the fibers were determined. It was shown that the average fiber diameter increased with adding of the NR content, while the linear and surface densities changed slightly. Using the differential scanning calorimetry, the thermal characteristics were obtained. It was found that the glass transition temperature of polylactide increased by 2–5 °C, and the melting temperature increased by 2–4 °C in the presence of natural rubber in the samples. By the method of electronic paramagnetic resonance at T = 50 and 70 °C it was determined that the mobility of the amorphous phase in PLA/NR fibers increased with the addition of NR. The adding of NR at a content of 15 wt.% increased the value of elongation at break by 3.5 times compared to pure PLA.

## 1. Introduction

In recent decades, the state of the environmental situation has led to the introduction of “green” polymers and composites based on them in various industries [1,2,3]. Film and fiber materials based on various polymers are finding new applications in medicine and the agricultural sector [4,5,6].

In this work, samples of non-woven fiber material were obtained by electrospinning. On the one hand, electrospinning is an economical, efficient, and versatile method for manufacturing fiber materials with nano- and submicron fiber diameters [7,8,9]. On the other hand, it is a complex process involving the hydrodynamics of weakly conducting Newtonian liquids and phase transformations—The evaporation of the solvent and the removal of the polymer fiber.

Non-woven mats obtained by electrospinning have a high porosity, small pore size and a good interconnected pore structure, which facilitate the transport of air molecules and the capture of particles [10,11]. Based on these characteristics, many types of non-woven fiber membranes were manufactured and studied [12,13,14]. Modern trends in the development of innovative fiber are focused on non-toxic, eco-friendly materials [15,16]. The use of a thermoplastic biodegradable polymer—polylactide (PLA) obtained from plant-based renewable raw materials, in large-scale production has become possible due to the properties of PLA, which can serve as the basis for materials that are competitive with composites based on polyolefins and synthetic polyesters [17,18]. At the same time, the use of PLA for the creation of fiber and film composite materials reduces the dependence on oil [19].

The production and properties of non-woven fibers from PLA and PLA composites are described in [20,21,22]. Casasola and coauthors studied the effect of solvent and solvent mixtures on the morphology and diameter of PLA nanofibers. As a result, it was found that the boiling point, conductivity, and viscosity of the solvent affect the process of forming fibers, their structure, and properties [20]. Earlier work by Jun (2003) also showed the influence of the solvent on the process of electrospinning polylactide fibers, on the morphology and diameter of the elementary fiber [21]. The authors [22] obtained and studied non-woven PLA/PHB fibers and determined their antimicrobial ability. Intensive growth of *Chaetomium globosum* and *Aspergillus niger* was detected on untreated PLA/PHB fibers, which was quite expected since *Aspergillus niger* is one of the most aggressive micromycetes [23].

Despite a significant number of articles on the morphology, thermal and mechanical properties, degradation of non-woven PLA fibers and composites based on it [24,25,26], there are no reports of the production and properties of non-woven fiber PLA/NR besides the work [27]. There are some papers that investigate the blends of PLA/NR obtained by dynamic vulcanization [28] or PLA/ENR [29], PLA/NBR [30], PLA/NR prepared by a melt mixer [31].

This work is devoted to obtaining and studying the morphology and properties of non-woven PLA-NR fibers. Natural rubber is an elastomer obtained from a renewable source of raw materials, from rubber tree juice. NR combines biocompatibility, biodegradability, and environmental friendliness. Addition of NR to the PLA matrix will improve the elasticity of the polylactide and produce a bio-material.

## 2. Materials and Methods

### 2.1. Sample Preparation

Poly(lactic acid) PLA, 4032D (with about 2% of D-lactide) with molecular weight (M_w_) of 1.7 × 10^5^ g/mol and ρ = 1.24 g/cm^3^ was procured from Nature Works (Minnetonka, MN, USA) and used without any purification. Natural rubber (NR), SVR-3L with Mooney viscosity 50 ± 5 (100 °C) and poly(cis-1,4-isoprene) content: 91–96, wt.% was kindly supplied by Vietnam Rubber Group (Ho Chi Minh City, Vietnam).

The polymer solutions for electrospinning were prepared by dissolving PLA and PLA/NR in the right ratio in 100 mL of chloroform. The mixtures were heated at 60 °C for about 4–5 min. The polymer solution was placed in a syringe with a needle inner diameter 0.7 mm, set up vertically. The electrospinning experiments were performed at room temperature (20 ± 2 °C). The consumption of the solution was (9–11) × 10^−5^ g/s. The sample weight was 9 g per 100 mL and the ratio of the components (PLA:NR, wt.%) was 100:0, 95:5, 90:10 and 85:15. The electrospinning process was run for at least 5 h with a voltage of 17.5–19 kV, a distance of 17 ± 1 cm between the needle tip and the collector until a non-woven fiber was produced. Five fiber samples were prepared for each solution.

### 2.2. Analysis of Crystallization

Thermal analysis was performed by differential scanning calorimeter (DSC) using a DSC 204 F1 device (Netzsch, Selb, Germany) under a nitrogen atmosphere. Samples of about 5.0–5.4 mg sealed in aluminum pans were heated from room temperature to 200 °C at rate of 10 °C/min. Indium with T_m_ = 156.6 °C was used as a calibrant. The crystallinity of PLA (χ_c_) was estimated from the first heating cycle using the following Equation (1):

χ_c_ (%) = 100% × (Δ*H*_m_/Δ*H*_m_*) (1)

where Δ*H*_m_ is the enthalpy of melting during heating, Δ*H*_m_* is the enthalpy assuming 100% crystalline PLA homopolymer 93.1 J/g [1].

### 2.3. Determination of Mechanical Characteristics

Mechanical characteristics were determined according to ISO 527-4: 2012 by tensile compression testing machine Devotrans DVT GP UG 5 (Istanbul, Turkey). The tensile strength, elongation at break, and modulus of elasticity were determined. The crosshead velocity was 0.5 mm/min. The data was averaged by seven samples.

### 2.4. Fiber Structural Characteristics and Morphology

The structural characteristics of the fiber samples were determined according GOST 15902.2-2003 (ISO 9073-2:1995): Nonwoven fabrics. Methods of determination of structural characteristics.

The morphology of electrospun PLA and PLA/NR fibers was characterized by scanning electron microscopy Philips SEM-500 (Eindhoven, The Netherlands).

### 2.5. Electronic Paramagnetic Resonance

The molecular mobility was studied by the spin probe method on an EPR-V automated EPR spectrometer (Semenov Federal Research Center For Chemical Physics, Russian Academy of Sciences, Moscow). The stable nitroxide 2,2,6,6-tetramethylpiperidine-1-oxyl was used as a probe. The radical was introduced from vapors in the films at 40 °C to a concentration of up to 10^−3^ M. The EPR spectra were recorded in the absence of saturation, which was checked according to the dependence of the signal intensity on the microwave field power. The probe rotation correlation time (τ_c_) was calculated from the EPR spectra according to the Equation (2) [24]:

τ_c_ = ∆H_+_ × [(I_+_/I_−_)^0.5^ − 1] × 6.65 × 10^−10^ (2)

where ΔH_+_ is the width of the low-field component of the spectrum, and I_+_/I_−_ is the intensity ratio of low- to high-field components, respectively.

### 2.6. FTIR Spectroscopy

The IR spectra were recorded on a Perkin Elmer Spectrum 100 FTIR spectrometer (USA) at temperature (22 ± 2) °C.

### 2.7. Statistical Processing

The experimental results were calculated as the arithmetic mean and its standard error. The calculations were performed using Statistica 8.0 software (Dell Software Inc., Round Rock, TX, USA) and Microsoft Excel 2007.

## 3. Results

### 3.1. Morphology and Structural Characteristics

**Figure 1:**
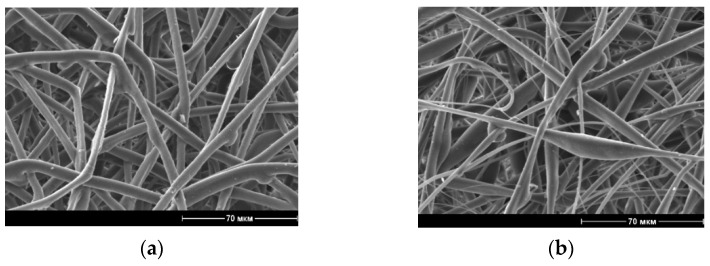

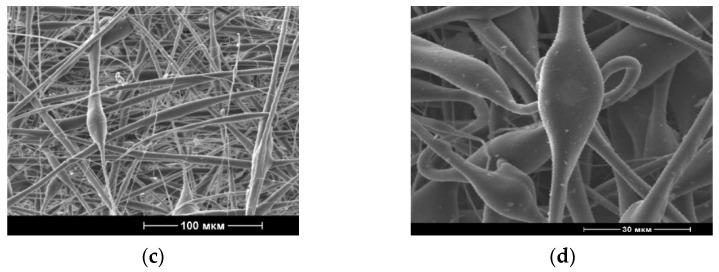
Scanning electron micrographs of PLA (**a**) PLA/NR fibers with 5 wt.% (**b**) 10 wt.% (**c**) and 15 wt.% (**d**) of NR content.

Non-woven fiber materials, PLA/NR with the different contents of NR in the spinning solution, were obtained by electrospinning. Figure 1 shows the scanning electron micrographs of the PLA and PLA/NR samples.

The obtained composites are heterophasic and while forming and evaporating the solvent the system stays in a non-equilibrium state, which leads to the appearance of different fiber thicknesses (Figure 1b–d). With an increase in a NR content in the PLA matrix, the fiber structure tends to bead-string morphology. The difference in the viscosity of PLA and NR also affects the formation of beads. So, the dynamic viscosity of the PLA is 0.52–0.54 Pa·s, and those of PLA/NR containing 10 wt.% of NR is 1.4 times higher.

Important characteristics of fiber materials are linear and surface density, as well as the average diameter (*d_av_*) of the fiber. A large role is played by the solvent. There are works that show the effect of the solvent on the formation of non-woven fibers [20,21]. In the case of a two- or more-component system, the process of selecting a solvent becomes more complicated. Both PLA and NR are well dissolved in chloroform, and the chloroform evaporates quickly enough to allow the fibers to harden until they reach the collector, but not too quickly to allow maximum pulling of the fibers until they harden. The average diameter of the fiber (Table 1) increases with the addition of NR to the PLA matrix, but the linear and surface density change slightly, so when the NR content is 15 wt.% the linear and surface density values increase by 3%, which is within the error range.

### 3.2. DSC Data

**Figure 2:**
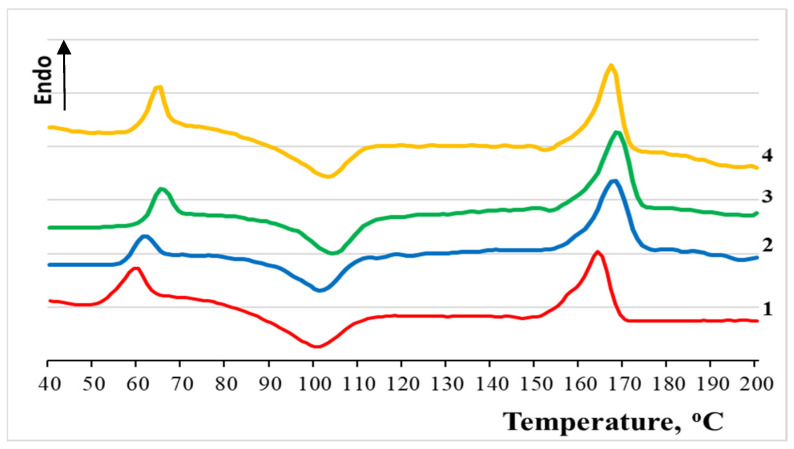
Melting thermograms of PLA/NR fibers. The content of NR, wt.%: (1) 0; (2) 5; (3) 10; (4) 15.

The method of differential scanning calorimetry was used to determine the thermophysical characteristics of PLA/NR non-woven fibers. Melting thermograms are shown in Figure 2. One can see that the adding of NR into the PLA matrix changes the temperature of the phase transitions: T_g_, T_cc_ and T_m_ of the thermoplastic.

Numerical data is shown in Table 2. Thus, the glass transition temperature (T_g_) of PLA increases by 2–5 °C with an increase in the content of NR in the spinning solution. A similar relationship is observed for the melting temperature, the degree of crystallinity (χ_c_) of the PLA changes by 2–4%, and the crystallization temperature has an increasing trend. The change in the T_g_ of the polylactide may be a consequence of a change in the supramolecular structure, and the influence of differences in the thermal expansion coefficients of the mixed components is not excluded [32]. The small changes in T_m_ can be attributed to possible reorganization in the crystalline phase during heating. In the process of cold crystallization, the completion of crystallites probably occurs, and natural rubber facilitates the process of PLA crystallization.

It is important to note that Chi [31] and coauthors obtained a similar result with respect to the degree of crystallinity in the mixed compositions obtained from the melt, and scientists from Thailand [33] found a significant increase in the degree of crystallinity of PLA with an NR content of 10–30 wt.% in mixtures obtained from the melt.

### 3.3. FTIR Spectroscopy

**Figure 3:**
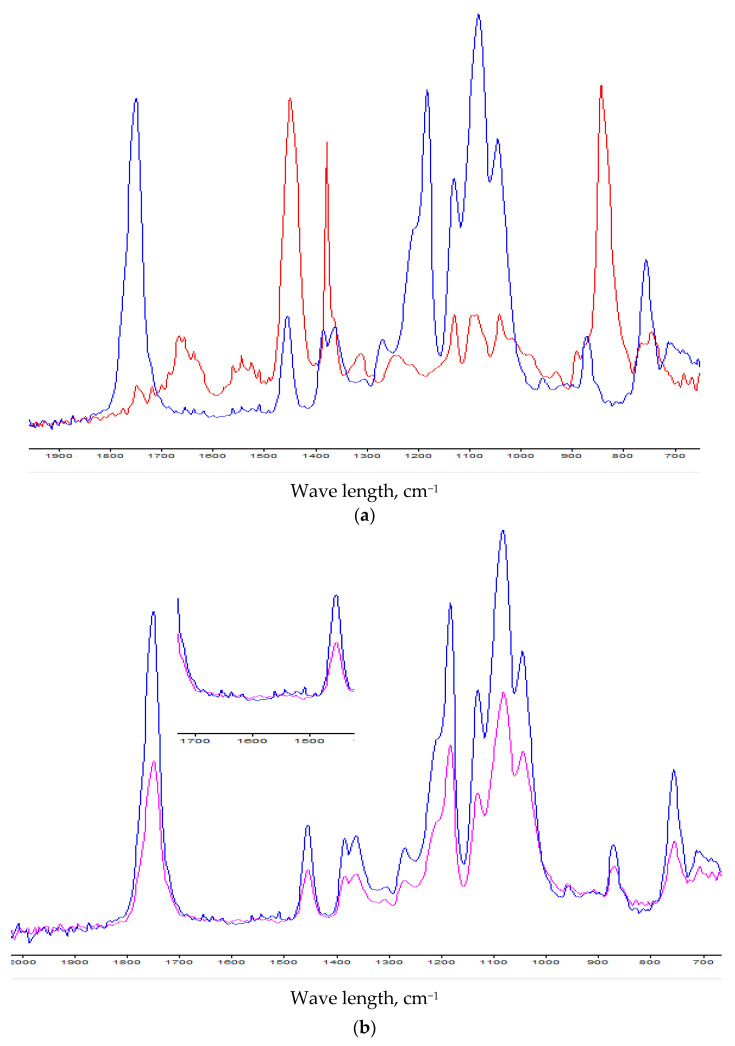
(**a**). IR-spectra of pure NR (red) and PLA/NR with 15 wt.% NR content (blue). (**b**). IR-spectra of pure PLA (pink) and PLA/NR (blue) with 15 wt.% NR content (blue).

IR-spectra of pure NR and PLA/NR (with 15 wt.% NR content) and pure PLA and PLA/NR (with 15 wt.% NR content) are shown in Figure 3a,b respectively. The spectra of all fiber samples are identical. For clarity, the PLA/NR spectrum with an NR content of 15 wt.% is shown.

Comparing the spectra of pure polymers and PLA/NR, it can be seen that on the PLA/NR spectrum bands appear in the area of 1660–1640 cm^−1^ and 1550–1520 cm^−^^1^ (the small insert in Figure 3b). These bands correspond to the valence vibrations of C=C– groups in natural rubber [34]. Also on the spectra of pure PLA and pure NR there is a band of 755 cm^−^^1^. In the PLA spectrum, it refers to stretching vibrations of –C–C– bonds in the polylactide [35]. In the PLA/NR spectrum the intensity of this band increases. It means that the NR contributes to the intensity of this band. Thus, the structure of the polylactide changes with the addition of NR, which affects the thermophysical characteristics (Table 2), as well as the dynamic and mechanical properties that are presented below.

### 3.4. Nishi-Wang Equation

From the DSC data, it follows that during electrospinning from a solution, NR affects the structure formation of the PLA. The effect of the amorphous component of the mixture on the PLA melting temperature can be analyzed based on the Nishi-Wang Equation (3) [36].

T_m_/T_m_* = 1 + B (ν_2_/Δ*H*_m_*) × φ_2_^2^ (3)

where B = δ_12_RT/ν_2_ at T = T_m_*—melting point of 100% PLA, measured under the same heating conditions as for mixtures; ν_1_ and ν_2_—molar volumes of PLA and NR, respectively; Δ*H*_m_*—heat of melting of 100% PLA crystal; δ_12_—compatibility parameter; φ_2_—volume fraction of NR; R—universal gas constant.

**Figure 4:**
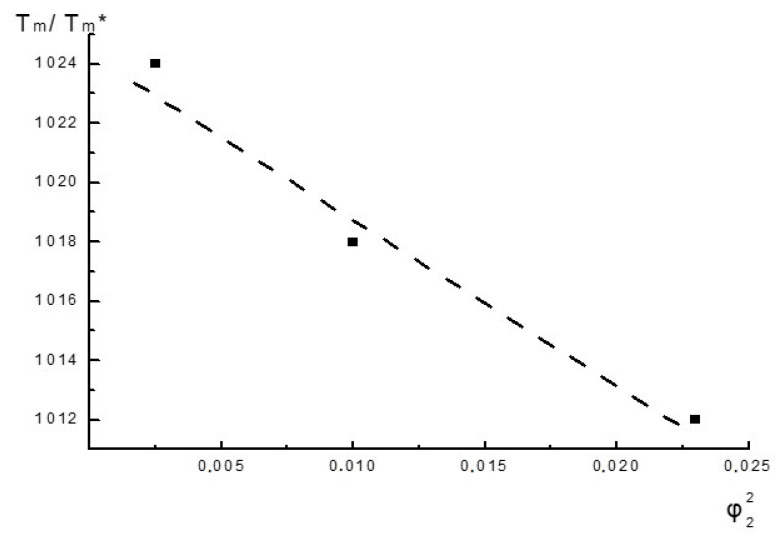
The Nishi-Wang parameter for PLA/NR fibers.

In the work, the compositions were measured under the same scanning conditions without determining the equilibrium values of the T_m_. According to Equation (1), the dependence of T_m_/T_m_* on φ_2_ is shown in Figure 4.

This dependence is of a qualitative nature, but the change in T_m_/T_m_* is actually described by Equation (1). The Parameter B, and therefore δ_12_ are negative, which indicates some limited interaction at the level of the PLA and NR segments, which is probably the reason why there are changes in different characteristics.

### 3.5. Macromolecules’ Dynamic

**Figure 5:**
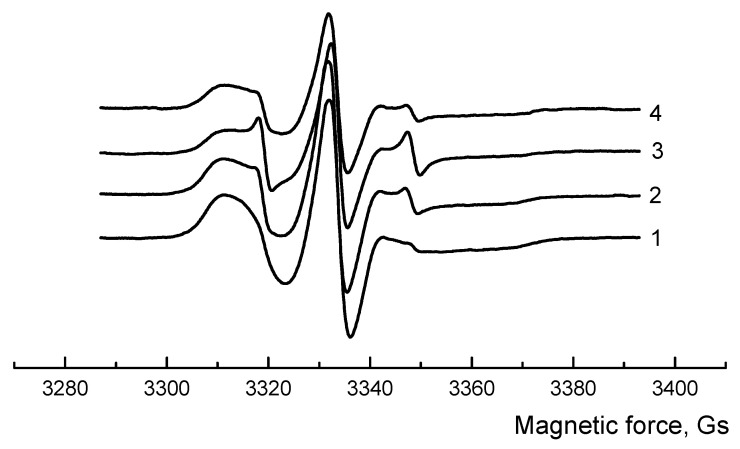
EPR spectra of PLA/NR fibers. The content of NR, wt.%: (1) 0; (2) 5; (3) 10; (4) 15. The radical was inserted at 70 °C.

The EPR method was used to estimate the macromolecular mobility of the amorphous phase in fiber composites. The radical probe was introduced at two temperatures, at T = 50 °C, when the PLA is in a glassy state, and above the PLA glass transition temperature—at 70 °C (Figure 5).

Data is presented in Table 3. One can see that at 50 °C, the correlation time (τ_c_) in PLA-NR samples is slightly lower than at 70 °C. Changes in the obtained values are small, since the correlation time at 50 °C is determined by the prevailing phase, and the PLA is in a glassy state and the mobility of macromolecule segments is small, despite the presence of an amorphous component—natural rubber.

If the probe is inserted at 70 °C, a different situation can be observed. Above the glass transition, the segments of the PLA matrix are more mobile, in addition, the increase in the proportion of the amorphous phase due to NR at this temperature is more evident. Also according to the IR spectra, the amount of the amorphous phase increases. In composites, in comparison with PLA, segmental mobility increases by 3.5–4 times. The results obtained by the EPR method are in agreement with the DSC data: the degree of PLA crystallinity increases in the presence of NR due to a raising in segmental mobility and, apparently, easier stacking of PLA chains in crystallites.

### 3.6. Mechnical Properties

**Figure 6:**
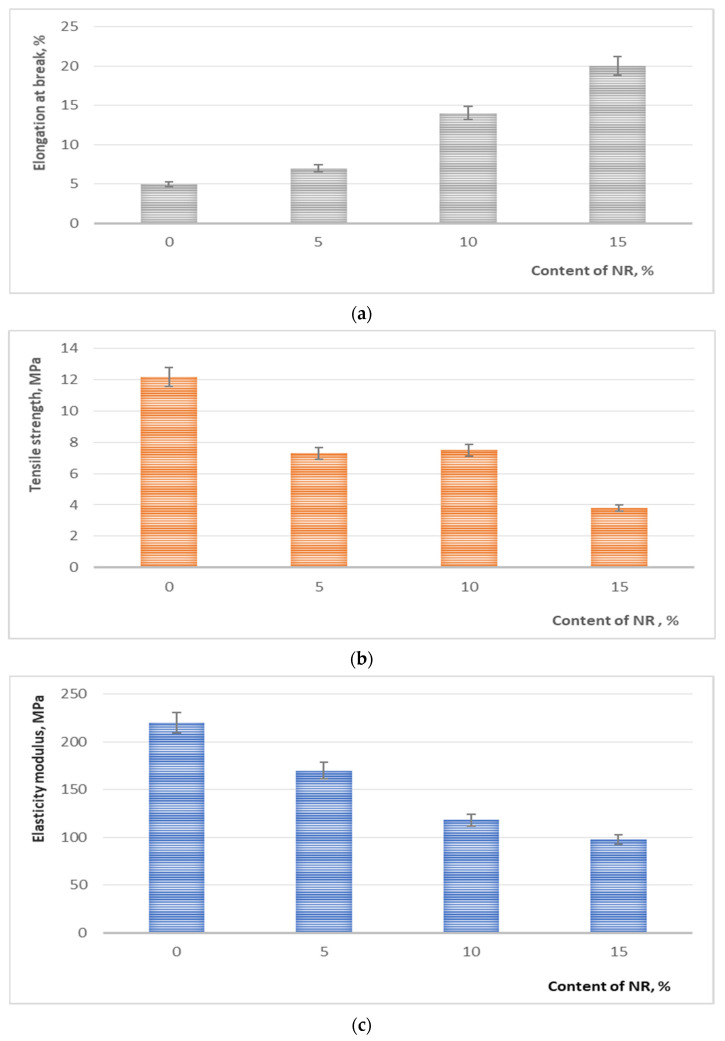
The dependence of the mechanical properties of PLA/NR fibers on the NR content: elongation at break, % (**a**); tensile strength, MPa (**b**) and elasticity modulus, MPa (**c**).

All questions related to the crystallization and morphology of a polymer or polymer composite ultimately affect the mechanical properties. The mechanical characteristics of non-woven fibers are shown in Figure 6. It is shown that with an increase in the content of rubber in the molding solution, the relative elongation at the break of the obtained fibrous materials increases (Figure 6a). With a content of 15 wt.% NR the value of *ε* increases by 3.5 times. The values of the tensile strength and elasticity modulus (Figure 6b,c) in PLA/NR fibers decrease with an increase of a NR content in non-woven fiber.

The PLA/NR system is a type of modification of a thermoplastic polymer by an elastomer. The mechanical properties of such composites do not change additively and are complex [37,38]. As a rule, the addition of rubber to the thermoplastic matrix leads to an increase in the elongation at break and a decrease in the tensile strength. The reduced PLA content in the composites and the poor interfacial adhesion between PLA and NR may be the reasons for the decreased the tensile strength and modulus. Increasing the rubber content to 50–70 wt.% phase inversion may be observed [31,37].

## 4. Discussion

The morphology and diameter of the resultant fibers depend on many parameters. When obtaining fibrous materials from a polymer solution by evaporation of the solvent, the system passes the concentration interval from the initial concentration of the solution to 100% concentration. The process of evaporation of the volatile solvent is non-equilibrium, the viscosity of the system changes and a different thickness of the fiber is formed. Selecting the optimal parameters of the electrospinning process such as the electric voltage, the distance between the capillary, and the electrode–substrate deposition, allowed to obtain samples of non-woven fiber materials of satisfactory quality. As is known, the electric voltage must correspond to the viscosity and surface tension of the polymer solution to ensure the formation and maintenance of a solution jet from the capillary. The solvent also plays an important role. The selection of the solvent is one of the main factors influencing the electrospinning ability and fiber properties. It is known that electrical conductivity and surface tension (should be less 0.050 N/m) of the solvent impact on the fiber diameter and morphology. However, the choice of solvent or solvent mixture may be complicated for a polymer-polymer system therefore in this work chloroform was chosen with the electrical conductivity: 1.0 × 10^−4^ μS/cm and the surface tension: 0.027 N/m.

The properties of a polymer blend depend on the properties of homopolymers, as well as on their macro- and microstructure, and their interface. Although PLA and NR are immiscible polymers, according to the Nishi-Wang equation some interaction between the PLA and NR phases occurs. As early as 1971, Helfand assumed that in the interphase region between immiscible polymers there is a certain degree of interpenetration, a limited diffusion of chain segments [39,40]. According to Kuleznev, the physical meaning of interfacial interaction is that the boundary macromolecules do not have the ability to occupy the same conformations as the macromolecules in the bulk of polymers [32]. The entropy of the boundary layers is lower. It is possible to transfer segments of macromolecules of a polymer into a layer of another one. The presence of interfacial interaction affects the thermophysical characteristics and various properties of the studied samples. The relationship between the dispersed phase (NR) and the dispersion medium (PLA) is important. The adding of an amorphous component (NR) into the matrix of a crystallizing polymer (PLA) will increase the proportion of the amorphous phase in the fiber composites, which in turn will necessarily affect the strength characteristics of the material. NR in the composites ensures the manifestation of forced elasticity, and the value of the elongation at break increases. The easier plastic deformation could be caused by different phenomena: cavitation (debonding of rubbery particles from PLA, cavitation inside the particles), or generation of crazes [38]. On the other hand, too large cavities could lead to premature fracture, which is not the case here.

The presented results support the necessity for the further investigation. It is planned to obtain PLA/NR fibers from a solution using a mixed-solvent system, as well as produce PLA/NR film samples, and compare the process of PLA crystallization in films with those in a fibrous material.

## 5. Conclusions

In the work PLA/NR fibers with an NR content of 0, 5, 10 and 15 wt.% were obtained by electrospinning, then their structural, thermal, dynamic and strength properties were studied. Due to the difference in the viscosity of polymers and the heterophase of fiber composites, a bead-string morphology is observed. Linear and surface densities do not depend much on the NR content in composites. According to the Nishi-Wang equation, some limited interface between PLA and NR was found. Adding of NR to the PLA matrix leads to an increase in the temperature of phase transitions (T_g_, T_cc_, T_m_) and a slight increase in the degree of crystallinity of the PLA. The EPR method shows a raising of mobility in the amorphous phase of fiber composites compared to pure PLA, and these changes are more noticeable when the experiment is conducted above the glass transition temperature of PLA. Mechanical properties depend on the composition of the PLA/NR fibers. Due to the forced elasticity, the elongation at break enhances, and since the amount of amorphous phase in fibrous samples increases, the tensile strength decreases with an increase of the NR content.

## 6. Patents

Tertyshnaya, Y.V.; Shibryaeva, L.S. Biodegradable fiber composite based on polylactide and its application for the plants growing, RU 2734883 C1, 2020, bul.30.

## Figures and Tables

**Table 1:** Structural characteristics of PLA/NR non-woven fibers.

NR, wt.%	Structural Characteristics		
	*d_av_*, µm	Linear Density, g/m	Surface Density, g/m^2^
0	5.84–7.20	0.950 ± 0.023	47.5 ± 0.89
5	6.24–8.22	0.963 ± 0.018	48.2 ± 1.16
10	6.51–8.81	0.955 ± 0.015	47.0 ± 1.03
15	6.64–9.24	0.977 ± 0.021	49.1 ± 1.20

**Table 2:** Thermophysical characteristics of PLA/NR non-woven fiber.

NR, WT.%	Thermophysical Characteristics			
	T_g_, °C (Δ ± 0.5 °C)	T_m_, °C (Δ ± 0.5 °C)	T_cc_, °C (Δ ± 0.5%)	χ_c_, % (Δ ± 1%)
0	61	164	101	34
5	63	168	102	36
10	66	167	104	38
15	65	166	104	37

**Table 3:** The correlation time of the probe in PLA/NR fibers. The radical was inserted at T = 50 °C and T = 70 °C.

NR, wt.%	The Correlation Time	
	τ_c_ × 10^−10^ c^−1^, 50 °C	τ_c_ × 10^−10^ c^−1^, 70 °C
0	4.7 ± 0.15	62.0 ± 0.23
5	3.8 ± 0.11	16.7 ± 0.20
10	3.3 ± 0.12	17.8 ± 0.18
15	2.9 ± 0.18	15.6 ± 0.15

## Data Availability

The data presented in this study are available on request to the corresponding author.
